# A Text-Book of Medical Treatment of Diseases and Symptoms

**Published:** 1901-01

**Authors:** 


A Text-Book of Medical Treatment of Diseases and Symptoms. For the use of students and practitioners of medicine. By Nestor Tirard, M. D., F. R. C. P., Professor of Principles and Practice of Medicine, King's College, London. Adapted to the U. S. Pharmacopeia by E. Quin Thornton, M. D., of Jefferson Medical College, Philadelphia. In one 8vo volume of 624 pages. Philadelphia and New York: Lea Brothers & Co [Price, cloth, $4.00 net.]

The books to which the busy practitioner most often turns are those which treat of the cure of disease, and these are the ones least liable to be found in his small neglected library. The demand for such works has stimulated writers to prepare such treatises, and among the best thus far produced Tirard's stands high on the list. To prepare such a work the author must have had large opportunities and great clinical advantages if the book is to be at all original, and these qualifications this author has to a marked degree. The information regarding the treatment of the various diseases is sound and has received general approval, and although incidentally newer remedies are frequently mentioned the author has included only those which appear likely to have a permanent place in practice. Many formulæ, adapted to the United States Pharmacopeia by Dr. Thornton, accompany the text.

The author is, perhaps, somewhat too conservative in the recommendation of drugs of recent origin and it would almost seem as if he were not quite up with the times. Too many new drugs of synthetical origin now find refuge in our practice, some of which are valuable, others worthless and foisted upon the profession by foreign manufacturers simply for commercial gain. The author has been quick to recognise this state of affairs and in his zeal to be trustworthy and reliable has omitted some of real value. The treatment is vividly described, the language plain and a book of such pretentions is sure to have a large sale.

The presswork and binding are excellent. W. C. K.

